# Natural genetic variation in photosynthesis: an untapped resource to increase crop yield potential?

**DOI:** 10.1111/tpj.14568

**Published:** 2019-11-13

**Authors:** Michele Faralli, Tracy Lawson

**Affiliations:** ^1^ School of Life Sciences University of Essex Colchester CO4 4SQ UK; ^2^ Department of Biodiversity and Molecular Ecology Research and Innovation Centre Fondazione Edmund Mach via Mach 1 38010 San Michele all’Adige (TN) Italy

**Keywords:** photosynthesis, genetic variation, wheat, yield, photosynthetic efficiency

## Abstract

Raising crop yield potential is a major goal to ensure food security for the growing global population. Photosynthesis is the primary determinant of crop productivity and any gain in photosynthetic CO_2_ assimilation per unit of leaf area (*A*) has the potential to increase yield. Significant intraspecific variation in *A* is known to exist in various autotrophic organs that represent an unexploited target for crop improvement. However, the large number of factors that influence photosynthetic rates often makes it difficult to measure or estimate *A* under dynamic field conditions (i.e. fluctuating light intensities or temperatures). This complexity often results in photosynthetic capacity, rather than realized photosynthetic rates being used to assess natural variation in photosynthesis. Here we review the work on natural variation in *A*, the different factors determining *A* and their interaction in yield formation. A series of drawbacks and perspectives are presented for the most common analyses generally used to estimate *A*. The different yield components and their determination based on different photosynthetic organs are discussed with a major focus on potential exploitation of various traits for crop improvement. To conclude, an example of different possibilities to increase yield in wheat through enhancing *A* is illustrated.

## Significance Statement

The MS focuses on natural genetic variation in photosynthesis and other physiological processes as possible targets for exploitation for yield improvements.

## Introduction

Photosynthesis is the primary determinant of crop productivity and any gain in photosynthetic efficiency has the potential to result in increases in yield (Flood *et al.*, 2011; Lawson *et al.*, 2012; Gu *et al.*, 2014). Free air CO_2_ enrichment (FACE) studies have provided substantial evidence that increased photosynthetic rates have translated into greater crop yields, demonstrating the link between photosynthesis and yield (Ainsworth and Long, 2005). The yield potential of a crop can be described by the following equation:

$$Y_{p}=Q\cdot \varepsilon_{i}\cdot \varepsilon_{c}\cdot \varepsilon_{p},$$

where *Q* is total solar radiation, *ε_i_* is the interception efficiency, *ε_c_* is the efficiency for conversion into biomass and *ε_p_* is the efficiency of partitioning biomass into harvested organs (Long *et al.*, 2015). In the absence of environmental stress, parameters such as harvest index are already close to the theoretical maximum (Foulkes *et al.*, 2010; Reynolds *et al.*, 2012). Additionally, many canopy traits such as canopy architecture (Long *et al.*, 2006), light interception (Murchie *et al.*, 2009), and photosynthetic duration (Shearman *et al.*, 2005) have been optimized. However, empirical analyses of the yield potential components demonstrate significant inefficiency in *ε_c_* in C_3_ crops (Zhu *et al.*, 2008, 2010), in which photosynthetic CO_2_ assimilation per unit leaf area (*A*) is the primary process (Kebeish *et al.*, 2007; Maurino and Peterhänsel, 2010; Walker *et al.*, 2016). The maximum potential conversion energy efficiency for C_3_ plants is 4.6% (Zhu *et al.*, 2010). However, plants including crops attain much less than this and therefore *A* is far from optimal and a target for further improvements (Parry *et al.*, 2010; Long *et al.*, 2015). Several studies have explored opportunities to increase energy conversion through genetic manipulation, including manipulating Calvin cycle activity (Simkin *et al.*, 2015; Lopez‐Calcagno *et al.*, 2018), RuBisCO properties (Parry *et al.*, 2003) and increasing the kinetics of non‐photochemical quenching for photo‐protection (Kromdijk *et al.*, 2016), all of which have translated into increased *A* and greater plant biomass. However, restrictions on growing genetically modified crops in many countries especially in Europe means that alternative methods to achieve increases in photosynthesis must be realized. An undervalued and currently unexploited opportunity to increase yield, not mutually exclusive of genetic engineering approaches, is the extensive natural variation in photosynthetic capacity in different C_3_ crops (Rawson *et al.*, 1983; Blum, 1990; Watanabe *et al.*, 1994; Fischer *et al.*, 1998; Hervé *et al.*, 2001; Pettigrew, 2004; Flood *et al.*, 2011; Gu *et al.*, 2012; Lawson *et al.*, 2012; Driever *et al.*, 2014; Gaju *et al.*, 2016; Carmo‐Silva *et al.*, 2017; Qu *et al.*, 2017; Pater *et al.*, 2017; Faralli *et al.*, 2019b). A number of studies have explored natural variation in photosynthesis in commercial wheat varieties (often relative to the year of release) (Fischer *et al.*, 1981, 1998; Blum, 1990; Watanabe *et al.*, 1994; Reynolds *et al.*, 2000; Xue *et al.*, 2002; Chytyk *et al.*, 2011; Sadras *et al.*, 2012), and demonstrated a correlation between photosynthesis and yield (e.g. Blum, 1990; Fischer *et al.*, 1998), although, such a relationship often depended on growth conditions (Xue *et al.*, 2002), or when measurements were taken during the growing season (Reynolds *et al.*, 2000), while others reported no relationship (e.g. Driever *et al.*, 2014). Variation in photosynthesis has been attributed to differences in radiation use efficiency (Sadras *et al.*, 2012), biochemical differences in RuBisCO activation properties (Carmo‐Silva and Salvucci, 2013), carboxylation efficiency (Driever *et al.*, 2014) and electron transport capacity (Carmo‐Silva *et al.*, 2017). In addition, variations in traits limiting the diffusion of CO_2_ to the site of carboxylation including mesophyll conductance (*g_m_*) (Jahan *et al.*, 2014) and stomatal conductance (*g_s_*) (Fischer *et al.*, 1998), which also includes the rapidity of *g_s_* responses to changing environmental conditions (Lawson *et al.*, 2010, 2012; Faralli *et al.*, 2019b) have been reported in several crops.

Here we review natural variation in physiological traits with a focus on: (i) photosynthetic capacity, which is determined by plant acclimatory responses and constrained by genetics; and (ii) dynamic short‐term modifications to *A* (e.g. biochemical factors such as the regulation of enzymes, *g_s_* and *g_m_*). The most common methods used to estimate *A* are evaluated and discussed. To conclude, we will focus on natural variation in *A*, stomatal physiology and the associated photosynthetic limitation in wheat (i.e. source limitation; lack of photo‐assimilates, or sink limitation) with a particular emphasis on the potential exploitation for crop improvement.

## Natural variation in photosynthesis

The biochemical processes of photosynthesis in C_3_ crops are considered essentially identical, (although recent metabolite profiling of C_3_ species by Arrivault *et al.* (2019) has reported considerable variation in levels of metabolites), however, significant intraspecific and interspecific variation in photosynthetic rates exists, providing a valuable source of unexploited genetic diversity (Flood *et al.*, 2011) (Table 1a). Furthermore, the physiological or genetic mechanisms underlying these differences in both photosynthetic potential as well as dynamic behaviour may provide valuable information on the performance of different cultivars under specific environments (Driever *et al.*, 2014).

**Table 1:** (a) Variation in crop photosynthesis rate per unit leaf area collected at saturating light and current atmospheric [CO_2_] (*A*_sat_). All the data were collected at 400 μmol mol^−1^ [CO_2_] while in Blum *et al.* (1990) and Fischer *et al. *(1998) measurements were taken at 345 μl L^−1^ [CO_2_], in Hervé *et al. *(2001) and Watanabe *et al. *(1994) measurements were taken at a [CO_2_] of 350 μmol mol^−1^ and in Gu *et al. *(2012) measurements were taken at a [CO_2_] of 380 μmol mol^−1^. (b) Variation in stomatal conductance (*g_s_*) and mesophyll conductance (*g_m_*) in different studies.

(a) Reference	Crop	Range of *A*_sat_ (μmol m^−2^ sec^−1^)	Intraspecific variation (μmol m^−2^ sec^−1^)	Relation with yield
Rawson *et al. *(1983)	Wheat	20–45 (mg dm^−2^ h^−1^)	12 mg dm^−2^ h^−1^	*A*_sat_ and yield not correlated. Cumulative CO_2_ fixation by flag leaf and yield *P* < 0.001 *r* ^2^ = 0.30
Blum (1990)	Wheat	9.6–16.6	7	High yielding cultivar showed highest *A* for the flag leaf
Watanabe *et al.* (1994)	Wheat	25.5–31.5	6	Yield data not present
Fischer *et al.* (1998)	Wheat	14.8–25.9	11.1	A*_sat_* and mean grain yield *r* 0.72 and 0.91 (*P* < 0.01)
Reynolds *et al.* (2000)	Wheat	20.9–27 at booting, 18–23.6 at anthesis, 23–11.8 at grain filling	11.2 to 5.6 depending on stage	*A*_sat_ and grain yield *P* < 0.01, *r* = 0.73
Chytyk *et al.* (2011)	Wheat	27.5–34.5	7	Yield data not present
Sadras *et al.* (2012)	Wheat	9.3–19.6	10.3	Data not plotted
Driever *et al.* (2014)	Wheat	30.5–19.1	11.4	Correlation between grain yield and *A* not significant (*P* > 0.05)
Carmo‐Silva *et al.* (2017)	Wheat	21.2–31.1 (pre‐anthesis), 17.1–23.7 (post‐anthesis)	9.9–6.6	*P* < 0.05 (*r* = 0.27 pre‐anthesis and *r* = 0.25 post‐anthesis)
Pettigrew (2004)	Cotton	20.3–37.7	17.4	Yield data not present
Pater *et al.* (2017)	Canola	5.5–22.5	17	Yield data not present
Gu *et al.* (2012)	Rice	12.8–25.5	12.7	Yield data not present
Hervé *et al. *(2001)	Sunflower	17.3 ± 10.2 (mean ± SD)	na	Yield data not present

(b) Reference	Crop	*g_s_* range (mol m^−2^ sec^−1^)	*g_m_* range (mol m^−2^ sec^−1^)	Note
Fischer *et al.* (1998)	Wheat	0.34–0.57	–	Field conditions
Jahan *et al.* (2014)	Wheat	–	0.51–1.05	Greenhouse conditions
González *et al.* (1999)	Barley	0.01–0.06 (cm sec^−1^)	–	Field conditions
Barbour *et al.* (2010)	Barley	0.25–0.52	0.05–0.50	Greenhouse conditions
Pater *et al.* (2017)	Canola	0.12–0.63	–	Large screening in greenhouse and field conditions
Hervé *et al. *(2001)	Sunflower	1.01 ± 0.08 (mean ± SD)	–	Greenhouse conditions
Ouyang *et al.* (2017)	Rice	0.15–0.31	0.05–0.21	Pot experiment

Intraspecific variation in photosynthetic traits in wheat and the potential effect of selection on photosynthesis was shown initially by Rawson *et al.* (1983) and then by Blum (1990) where breeding in Mediterranean environments had led to an increase in photosynthetic efficiency at saturating light for the modern cultivars compared with older varieties. Furthermore, Watanabe *et al.* (1994) followed by Fischer *et al.* (1998) phenotyped historical Australian and Mexican wheat cultivars for photosynthetic traits and reported a strong correlation between increased rates of CO_2_ assimilation and yield genetic gain with year of release, demonstrating that breeding has unintentionally selected for higher *A*. Subsequent research focusing on intraspecific variation in major crops such as cotton (Pettigrew, 2004), canola (Pater *et al.*, 2017), rice (Gu *et al.*, 2012), sunflower (Hervé *et al.*, 2001) and wheat (Reynolds *et al.*, 2000; Sadras *et al.*, 2012; Driever *et al.*, 2014; Carmo‐Silva *et al.*, 2017), highlighted a wide diversity of photosynthetic traits [including *A*_sat_ and the light and CO_2_ saturated rate of photosynthesis *A*_max_; the maximum carboxylation capacity *V*_cmax_ as well as the maximum rate of electron transport (*J*_max_)]. In addition, at the leaf level, CO_2_ uptake from the atmosphere to the site of carboxylation is subject to two main restrictions, stomatal and mesophyll, both of which therefore determine the rate photosynthesis. *g_s_* (the reciprocal of stomatal resistance) controls CO_2_ diffusion from the atmosphere into the intercellular air spaces in the gaseous phase (Farquhar and Sharkey, 1982; Sharkey, 1985). Subsequently, *g_m_* adds an additional limitation in the liquid phase for the diffusion of CO_2_ from the intercellular airspaces to the site of carboxylation in chloroplasts (Flexas *et al.*, 2008). Intraspecific variation exists for both *g_s_* and *g_m_* (Table 1b) in a series of food crops including wheat (Fischer *et al.*, 1998; Jahan *et al.*, 2014), barley (González *et al.*, 1999; Barbour *et al.*, 2010) and rice (Ouyang *et al.*, 2017). Therefore exploiting the existing natural variation in photosynthesis as well as optimizing the components determining *A* in elite cultivars (Driever *et al.*, 2014), landraces (Gaju *et al.*, 2016) and wild relatives (Prins *et al.*, 2016) could provide novel targets for crop improvement.

However, while Crosbie *et al.* (1981) showed that leaf photosynthesis of maize can be improved by recurrent selection (i.e. increasing the frequency of favourable alleles for quantitatively inherited traits, in this case for *A*) five cycles of recurrent phenotypic selection did not produce the expected results in term of productivity, and changes in grain yield were not significant for any of the populations tested (Crosbie and Pearce, 1982). Indeed, correlating photosynthesis with yield is not straight forward, with inconsistencies in the relationship described in the literature, for example positive correlation (Carmo‐Silva *et al.*, 2017), no correlation (Ojima, 1974; Driever *et al.*, 2014), or a correlation but only when photosynthesis was measured at particular phenological stages (Gaju *et al.*, 2016). These inconsistences in the relationship between *A* and yield emphasize the complexity of yield formation in crops that is based on a series of interrelated subcomponents (Miralles and Slafer, 2007), and that is further complicated by the different methodologies used to estimate *A* and the influence of fluctuating environmental conditions to which the crop is subjected (Lawson *et al.*, 2012). Individual point measurements of *A*_sat_ or *A*_max_ taken either at different times during the crop cycle or on individual leaves within the canopy, often do not correlate with yield (Rawson *et al.*, 1983; Driever *et al.*, 2014). Having said this, in some cases (i.e. Fischer *et al.*, 1998) a significant relationship between *A*_sat_ and some yield components (i.e. grain number) or the average grain yield (over 5 years) was evident. In addition, when operational *A* was measured (i.e. single measurements of *A* at light intensities similar to those experienced by the crop) in the field at the pre‐anthesis and post‐anthesis, a strong correlation with yield was reported (Carmo‐Silva *et al.*, 2017). Although an instantaneous ‘snapshot’ analysis of *A*_sat_ *,* carried out by Rawson *et al.* (1983) did not correlate with yield, a significant (*P* < 0.001) correlation between cumulative carbon assimilation of the flag leaf (measured as several snapshot *A*_sat_ measurements over the life cycle) and yield was observed. These studies highlight that the different methods used to measure *A*, the complexity of the relationship between *A*, plant growth and yield as well as the influence of the environment on these processes, need to be considered for estimating overall crop photosynthesis.

### Factors determining the variation in photosynthetic rate per unit leaf area

#### Biochemical factors and anatomical features

One of the first studies to examine the underlying biochemical function of interspecific variation in photosynthesis was Wullschleger (1993). Using response curves of *A* as a function of substomatal CO_2_ concentration (*A*/*C_i_*), Wullschleger demonstrated that most of the observed variation in *A* in the 109 species analyzed was attributed to variation in the underlying biochemistry and photosynthetic capacity with differences in both carboxylation capacity (*V*_cmax_) and electron transport capacity for RuBP regeneration (*J*_max_). Wullschleger (1993) also reported a positive correlation between *V*_cmax_ and *J*_max_ suggesting co‐ordinated regulation by these two processes. A small number of species (23) was reported to be limited by the utilization of triose phosphates, which ranged from 4.9 to 20.1 μmol m^−2^ sec^−1^, and reflects the short‐term interaction between *A* and starch–sucrose production, which ultimately reflects growth. It is clear from the representative *A*/*C_i_* curves in Wullschleger (1993), that the switch‐over point between limitation by carboxylation capacity and capacity for electron transport differed greatly in the four species illustrated, and that the maximum rates of *A* achieved were vastly different, which may be due to nitrogen allocation between RuBisCO and light harvesting. Nitrogen (N) concentration is a key determinate of *A*, as the majority of leaf N is invested in the photosynthetic apparatus, in particular RuBisCO (Hikosaka, 2010). Differences in N‐use efficiency and N concentration in different crops have suggested these as targets for both increased *A* and optimization of fertilization input (Guarda *et al.*, 2004; Hirel *et al.*, 2007). Although, there is evidence that within species variation in *A,* can be explained by differences in *V*_cmax _and *J*_max_ (Driever *et al*, 2014; Carmo‐Silva *et al.*, 2017), Driever *et al.* (2014) highlighted that the variation in carboxylation capacity was not due to RuBisCO content (or N allocation) but possibly RuBisCO activation, demonstrating further complexity in identifying specific targets for future wheat improvement. Furthermore, the same study also reported that some of the highest *V*_cmax_ values were found in older species, suggesting that photosynthetic capacity potential has not been fully exploited in past breeding programmes. However, since a major goal of future agriculture is to enhance resource‐use efficiency, it has been hypothesized that increasing RuBisCO carboxylation efficiency while reducing N allocation to RuBisCO might be a successful alternative in crops to improve or sustain *A* (Reynolds *et al.*, 2012). A reduction in RuBisCO content (up to 20%) led to a 10% lower N requirement in wheat, although reductions in *A* at high light intensities were also present (Reynolds *et al.*, 2012). More recently, Carmo‐Silva *et al.* (2017) found significant genotypic variation for RuBisCO carboxylation efficiency and RuBisCO content in wheat, with the cultivar Gatsby combining a high *A* and a low RuBisCO content, suggesting the potential of this preferable combination for further exploitation. Genetic engineering approaches have shown that increasing protein abundance (e.g. sedoheptulose1,7‐biphosphatase, SBPase) led to a significant increase in *A* which suggests that although photosynthesis requires a large number of protein–protein interactions, part of the genetic variation can be explained by differences in key protein abundance and activity, that result in improved photosynthetic capacity (Flood *et al.*, 2011; Simkin *et al.*, 2019) and which also might explain variation in metabolite profiles in C_3_ species (Arrivault *et al.*, 2019). The potential for exploiting natural variation in photosynthetic capacity has been demonstrated by Gu *et al.* (2014) who used a simulation analyses to assess the contribution that the natural variation in RuBisCO and electron transport rate could make to photosynthesis in rice and showed that exploiting this could increase rice yield by 22–29%, depending on location and year.

Many studies have focused on significant variation in photosynthetic capacity that is determined by acclimation to particular environmental conditions and genetically constrained. However, on a day‐to‐day basis, plants respond dynamically to changes in the surrounding environmental conditions that introduce a further layer of complexity to variation in photosynthesis as there is significant variation in dynamic responses. These dynamic processes include regulation and expression levels of enzymes (Sassenrath‐Cole and Pearcy, 1994; Hikosaka, 2010), dynamic regulation in response to environmental change (Sassenrath‐Cole *et al.*, 1994) including changes in non‐photochemical quenching of excess energy dissipating mechanisms (Külheim *et al.*, 2002; Lawson *et al.*, 2012), and the rapidity of stomatal responses (Lawson *et al.*, 2010, 2012; McAusland *et al.*, 2016) as well as developmental responses to growth environment (Flood *et al.*, 2011; Gilbert *et al.*, 2011).

Other processes, although not directly related to the photosynthetic machinery, also play a role in photosynthetic performance. For instance, sucrose transport from the mesophyll cells to heterotrophic tissues is of pivotal importance to sustain diurnal *A*, as it is generally accepted that *A* slowly decreases over the diurnal period due to the accumulation of photosynthates (Ainsworth and Bush, 2011). Recently, Ainsworth and Lemonnier (2018) reported the existence of genetic variation in different phloem loading mechanisms. Apoplastic loading‐unloading strategies are typically common in crop species and optimization cannot only help in sustaining *A* but also enhance sink strength, therefore these are potential targets to further maximize the diurnal integrated *A* (Ainsworth and Lemonnier, 2018). Furthermore, morphological factors substantially influenced *A* with long‐lived evergreen plants showing thicker leaves, with a higher leaf mass per unit leaf area, lower *g_m_* and therefore lower *A* than herbaceous plants (e.g. grasses) (Flood *et al.*, 2011). Therefore, differences in leaf functionality between species are the result of differences in leaf longevity and subsequent optimization of resource investment into photosynthetic organs.

#### Mesophyll and stomatal limitations of photosynthesis

Mesophyll conductance is considered a key trait for future improvement in *A* and yield potential, as lower resistance for CO_2_ diffusion to the chloroplast will allow higher substrate availability for carboxylation. Additionally, an attractive property of increasing *g_m_* is the potential to increase *A* without increasing water loss (Nadal and Flexas, 2018), which is not possible if *g_s_* is increased. *g_m_* can be dissected into three subcomponents: conductance through intercellular air spaces (*g*_ias_), through cell wall (*g_w_*) and through the liquid phase inside cells (*g*_liq_) (Flexas *et al.*, 2008; Terashima *et al.*, 2011). Variation in *g_m_* between species has been associated with alterations in all these components: for instance leaf structure may affect mostly *g*_ias_ and *g_w_*, in particular in thick leaves (Evans and von Caemmerer, 1996). In tobacco and soybean however, the most limiting component to *g_m_* appeared to be *g*_liq_ (Evans and von Caemmerer, 1996). The intraspecific variation in *g_m_* in crop species (Table 1) suggests that both morphological and metabolic factors are involved in CO_2_ diffusion into chloroplasts, with evidence of aquaporin modulation of the *g_liq_* component (Gillon and Yakir, 2000; Hanba *et al.*, 2004; Flexas *et al.*, 2006). For instance, overexpression of the aquaporin *OsPIP1;2* in rice increased *g_m_* by up to 150% compared with the wild type, resulting in greater biomass and yield (Xu *et al.*, 2018) and therefore provided evidence for a major role for aquaporins in the modulation of intracellular CO_2_ diffusion (Uehlein *et al.*, 2003; Uehlein *et al.*, 2008). Such studies often introduce the question ‘why have such changes not occurred naturally’, however it should be borne in mind that survival to reproduce is the plant’s ultimate goal, while photosynthesis and biomass may or may not be a part of this process, and therefore resource allocation and adaptive capacity will regulate such changes. *g_m_* is generally affected by both light and temperature, therefore *g_m_* can have a signficant impact on photosynthetic efficiency under fluctuating environments (e.g. Flexas *et al.*, 2008; Kaiser *et al.*, 2018). However, methodologies to quantify *g_m_* are time consuming and subject to high levels of uncertainty (see review by Pons *et al.*, 2009 and references therein), severely limiting high‐throughput phenotyping for this trait. In addition, *g_m_* is principally dependant on the physical capacity of CO_2_ to diffuse into the leaf tissue, and therefore dependent on *g_s_* and stomatal dynamics.

Increasing CO_2_ diffusion from the atmosphere to the leaf interior increases *A* (Lawson *et al.*, 2010) and it has been demonstrated in several studies that manipulating stomatal density (Tanaka *et al.*, 2013) or aperture (Lawson and Blatt, 2014; Duan *et al.*, 2015) increases *g_s,_* while recent studies have also suggested that stomatal kinetics and the rapidity of *g_s_* responses to the changing environment can increase carbon assimilation (McAusland *et al.*, 2016; Papanatsiou *et al.*, 2019). Increasing *g_s_* represents a trait already unintentionally included in breeding for high yielding varieties over many decades (Fischer *et al.*, 1998; Lu *et al.*, 1998; De Vita *et al.*, 2007). The positive effects of higher *g_s_* are numerous: in particular, under steady‐state conditions *A* is co‐related to *g_s_* and therefore high *g_s_* leads to elevated photosynthetic rates (by limiting the resistance to CO_2_ diffusion into intracellular airspaces) and, at the same time, increased evaporative cooling maintains optimal leaf temperature for *A* (Lawson and Blatt, 2014). As in C_3_ crops, a strong limitation of *A* is the temperature‐dependent increase in the oxygenation reaction of RuBisCO, the maintenance of optimal leaf temperature through high transpiration rates may be key in limiting photorespiration (Long *et al.*, 2006). In addition, although high *g_s_* may lead to early soil water depletion, it has been shown that the extra assimilates gained early in the growing season may enable greater carbon investment in roots (Blum, 2011), facilitating higher water extraction from the deeper soil layers therefore avoiding drought stress (Venuprasad *et al.*, 2011). It is therefore unarguable that *g_s_* is a key trait for improving crop yield potential and stability with substantial natural variation known to exist (Faralli *et al.*, 2019a). Stomatal conductance is determined by the number of stomata per unit leaf area and the pore aperture (which is often dependent on the size of stomata) both of which represent breeding targets for altered *g_s_*. For example, Arabidopsis lines lacking the epidermal patterning factor (EPF) 1 and 2, exhibited high stomatal density, greater *g_s_* and *A* when compared with the wild type Col‐0 (Franks *et al.*, 2015). Large natural variation in *g_s_* has been shown in a number of plants, including crops (i.e. Tichá, 1982; Roche, 2015; Faralli *et al.*, 2019a and Table 1), suggesting *g_s_* as a potential target to exploit for increased *A*, and therefore yield. Stomata open and close in response to changes in environmental cues (i.e. water availability, light, VPD) and depend upon plant hydraulic capacity, which is the plant’s ability to take up and distribute water around the plant (Sack and Scoffoni, 2013; Lawson and Blatt, 2014). In the field and inside a crop canopy, light and VPD can vary within minutes or even seconds. Stomatal responses are an order of magnitude slower than the response of *A*. For example, *g_s_* in wheat can take between 5 and 15 min to reach steady state following a shade or sun fleck (Faralli *et al.*, 2019b) and this lag in behaviour can limit *A* by up to 15%. Both intraspecific and interspecific variation have been shown to exist for stomatal rapidity (McAusland *et al.*, 2016; Faralli *et al.*, 2019b). In addition significant developmental effects on stomatal responses were shown by Faralli *et al.* (2019b **)**, in which a decrease in stomatal rapidity was reported in wheat during the post‐anthesis stage compared with the early booting stage. Therefore, *g_s_* and the dynamic response of *g_s_* can be potential unexploited targets for future crop improvement.

#### Measuring photosynthesis

**Figure 1:**
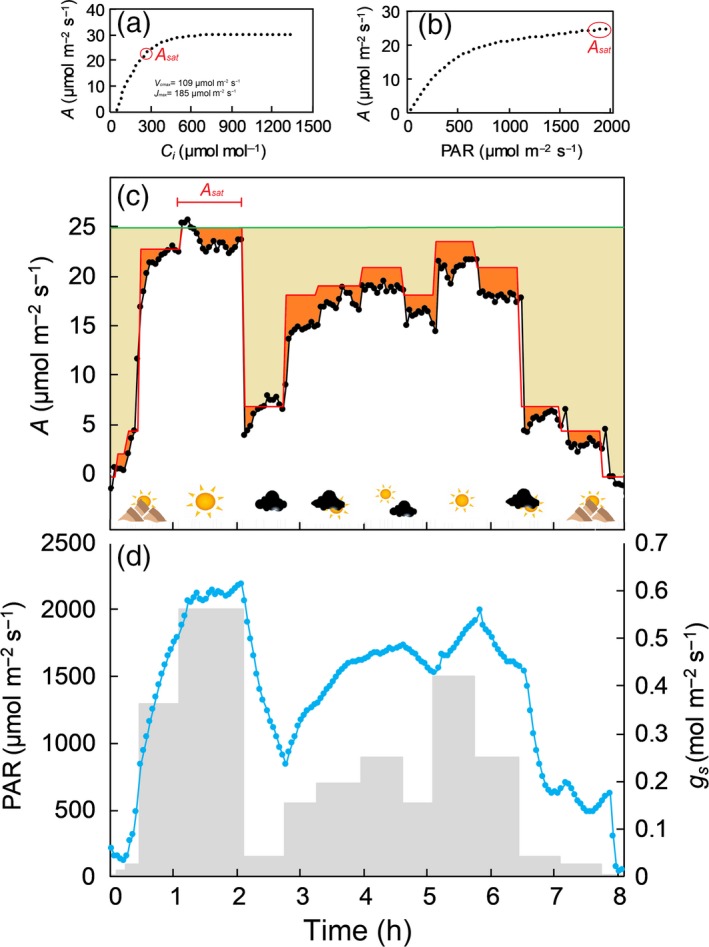
Example of a CO_2_ assimilation as a function of substomatal CO_2_ concentration curve (*A*/*C_i_*) and light (*A*/*Q*) (a, b respectively) measured on the flag leaf of wheat (cv. *Robigus*) at booting stage with an infrared gas analyzer (Li‐Cor 6400, Li‐Cor, USA). The *A*/*Ci *was measured at saturating light [1500 µmol m^−2^ sec^−1^ photosynthetic active radiation (PAR)] and a leaf temperature of 20°C. RuBisCO carboxylation efficiency (*V*_cmax_), the maximum electron transport rate for RuBP regeneration (*J*_max_) was estimated following Sharkey et al. (2007) and *A*_sat_ represents the light saturated *A* at current ambient [CO_2_]. (c, d) Diurnal measurement of photosynthetic CO_2_ assimilation (*A*) and stomatal conductance (*g_s_*) were measured on the same day as the *A*/*C_i_* and *A*/*Q* analysis on an adjacent part of the flag leaf at 20°C leaf temperature following a fluctuating light environment. In (c), black dots represent recorded *A* values, whereas the red line represents *A* estimated through the *A*/*Q* response. Orange regions represent the discrepancy in *A* between observed and modelled values from the *A*/*Q*. Light brown regions represent the potential overestimation in daily CO_2_ uptake if *A*_sat_ is used to assess total daily photosynthesis for plants growing under a natural fluctuating light regime. In (d), grey regions represent the light regimes (therefore the photosynthetically active radiation, PAR) at which the diurnal measurement with the Li‐Cor was carried out (following a simulated sunny‐cloudy pattern shown in (c)), while blue dots represent *g_s_.*

To date, most photosynthetic measurements have been based on two approaches using infrared gas analyzer systems: (i) capacity measurements where photosynthetic CO_2_ assimilation is measured as a function of substomatal CO_2_ concentration curves (*A*/*C_i_*) or as a function of light intensity (*A*/*Q*); or (ii) ‘snapshot’ or instantaneous measurements of *A* at selected times of the day. Additionally, other methods such as carbon isotope discrimination has been successfully used to estimate transpiration efficiency (Rebetzke *et al.*, 2002) and the photosynthetic contribution of different non‐foliar organs to grain yield (Sanchez‐Bragado *et al.*, 2016). In general, *A*/*C_i_* analysis is a powerful tool from which the biochemical properties under light saturated conditions, a constant leaf temperature and minimal boundary layer resistance can be determined. These conditions, necessary to assess maximum photosynthetic capacity are unlikely to represent those to which a leaf is exposed in the field (Lawson *et al.*, 2012; Driever *et al.*, 2014) (Figure 1). Assessing photosynthesis as a function of light (*A*/*Q* analysis) might be considered more representative of field conditions. These measurements can be used to model *A* rates over the diurnal period if incident light is monitored. However, it should be noted that *A/Q* curves are usually performed in near optimal environmental conditions, particularly at low vapour pressure deficits and often measured early in the diurnal cycle, both of which promote high *g_s_.* Therefore dynamic stomatal behaviour in the field environment could significantly decrease realized *A* when compared with the ‘theoretical maximum’ (Lawson *et al.*, 2012) (Figure 1c). Indeed, in a study on the effect of dynamic light on Arabidopsis by Vialet‐Chabrand *et al.* (2017) continuous diurnal gas exchange measurements of *A* were compared with those determined from *A*/*Q* response curve and incident photosynthetic active radiation (PAR), the latter failed to accurately predict the measured photosynthetic rates due to the limitation imposed by stomata (Figure 1c,1) as well as the late‐diurnal negative feedback on *A* (Vialet‐Chabrand *et al.*, 2017; Matthews *et al.*, 2018). Similar methodological drawbacks are present for simpler (and quicker) analysis of instantaneous or ‘snapshot’ measurements of photosynthesis that are either captured under natural irradiance, or use a light source to mirror *in situ* irradiance intensities. Stomatal limitation, enzyme activation states and photoinhibition can greatly influence short‐term photosynthesis. Additionally the environmental conditions that the plants have been exposed to before measurements also impact on instantaneous measurements, therefore increasing the complexity for data interpretation (Lawson and Weyers, 1999).

Therefore, it is not surprising that *A* is not always correlated with yield as analyses are often based on either photosynthetic capacity (e.g. *A*/*C_i_* and *A*/*Q* curves) that are not realized in the field or instantaneous measurements that represent a single point measurement of *A* that fails to characterize the diurnal photosynthetic pattern. In Table 1, the best link between yield and photosynthesis was found when integrated CO_2_ uptake was determined over the growing season or ‘operational’ photosynthesis was measured *in situ* (Rawson *et al.*, 1983; Carmo‐Silva *et al.*, 2017 respectively) suggesting that: (i) the different components defining yield are determined over spatial and temporal‐specific phenological stages, and therefore *A*_sat_ (the most used trait estimated in the literature) may correlate to a particular yield component rather than overall grain yield; (ii) *A_sat_* is representative of a steady‐state and optimal condition that crop plants hardly ever experience  in the field, and more realistic conditions for the analysis (e.g. subsaturating light intensities) are the most appropriate way to evaluate the realized *A* in natural dynamic environments; and (iii) although technically challenging, time consuming and subject to a high degrees of errors (e.g. time of senescence initiation), integrated CO_2_ uptake of the most photosynthetically active leaf (i.e. flag leaf) has the potential to be a representative trait linked to grain yield, at least in wheat (Rawson *et al.*, 1983). Therefore, new instrumentation that would enable diurnal and seasonal measurements of *realized* photosynthesis to be captured under natural dynamic field conditions and at different layers within the canopy is required (e.g. Salter *et al.*, 2018; Murchie *et al.*, 2018; Vialet‐Chabrand and Lawson, 2019). For example, the development of the ‘OCTOflux’ system by Salter *et al.* (2018), which is a multiplexed semiportable gas exchange system that enables *A*_max_ to be measured in eight leaves simultaneously. Furthermore, new tools are needed to facilitate high‐throughput measurements of photosynthetic capacity *in situ* and on large numbers of plants, such as the recent developments in hyperspectral imaging to rapidly measure *V*_cmax_ in the field (ca. 10 sec) (Meacham‐Hensold *et al.*, 2019). Although the approaches mentioned above have made significant advancements in measuring photosynthetic capacity, further developments on instrumentation are necessary to enable diel operational or realized photosynthetic rates to be determined, that are subject to the limitations driven by the growth conditions as well as the kinetics of various processes that a plant is subjected to over the dynamic diurnal period.

## Exploiting natural variation in photosynthetic capacity and stomatal function for improving crop productivity: a case in wheat

In wheat, several physiological traits have been unintentionally selected for to produce high yielding cultivars with increased grain number m^−2^ (GN) and hence yield (Fischer *et al.*, 1998). In the last few years, however, yield has stagnated in many countries suggesting the need for greater effort and new targets for increasing productivity (Ray *et al.*, 2012). The critical and source‐limited phase of stem extension determines GN (Slafer *et al.*, 2015). Two not mutually exclusive possibilities have been proposed to increase GN in wheat: (i) lengthening the duration and rate of growth and (ii) increasing resource availability (i.e. photosynthesis) (Miralles and Slafer, 2007). Indeed, increasing sedoheptulose1,7‐biphosphatase activity increased flag leaf photosynthetic capacity and GN per spike in greenhouse‐grown wheat (Driever *et al.*, 2017), suggesting that elevated flag leaf *A* can increase spike fertility. Most of the work characterizing photosynthesis in wheat has focused on flag leaf *A*, however, understanding and assessing earlier canopy photosynthetic efficiency (e.g. early stem extension) might be of greater importance to optimize spikelet and floret fertility. Several studies have already reported significant variation in photosynthetic capacity and light saturated rate of photosynthesis in wheat, suggesting the potential exploitation of diversity for selection and/or gene discovery (Driever *et al.*, 2014; Carmo‐Silva *et al.*, 2017). In particular, high‐throughput phenotyping approaches can help detect important genomic regions for leaf and/or canopy photosynthetic traits in wheat and speed up the selection of desirable traits. Either large panels of wheat with unknown ancestry or bi and multiparental populations (for quantitative trait loci analysis) can be used for this approach, as already demonstrated in rice (Teng *et al.*, 2004; Gu *et al.*, 2012) and recently reviewed by van Bezouw *et al.* (2019). In addition the development of single‐nucleotide polymorphism platforms in wheat (Wilkinson *et al.*, 2012) and the recently annotated genome of bread wheat (Appels *et al.*, 2018) will ensure a more comprehensive understanding of the genetic control of photosynthetic traits or other *A*‐determining traits such as *g_s_* and stomatal dynamics.

**Figure 2:**
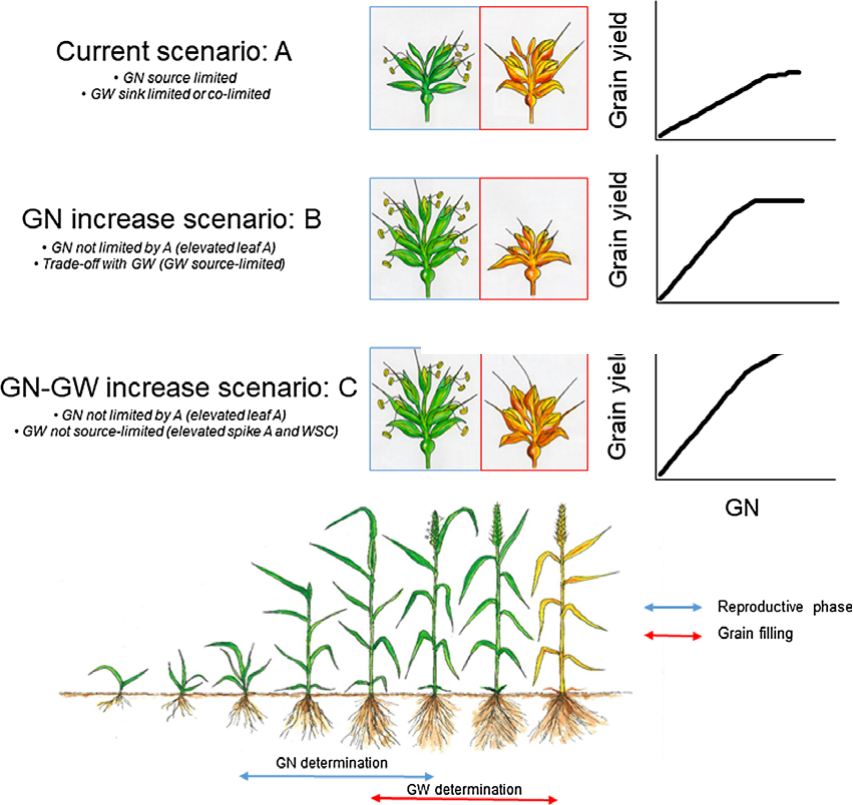
Theoretical scenarios for improving grain yield in wheat. (a) Current scenario with GN source‐limited and GW sink‐limited or both co‐limited. Here, grain yield is limited by GN. (b) Optimization of resources for grain number (GN) leads to a trade‐off with the individual grain weight therefore plateauing grain yield through the progress in GN. (c) Removal of source limitation is required for the reproductive and grain filling stages through optimization of flag leaf photosynthesis, spike photosynthesis and WSC remobilization, leading to a reduced trade‐off with the individual grain weight and therefore increase in grain yield.

As yield generally plateaus at high GN due to the trade‐off with grain weight (GW) (Figure 2, scenarios a and b) (Gambín and Borrás, 2010; Quintero *et al.*, 2018), understanding and potentially optimizing the GW component is of major importance for wheat yield improvement. Recent work reported the presence of a potential source limitation during grain filling (Álvaro *et al.*, 2008; Xie *et al.*, 2015; Quintero *et al.*, 2018). These reports suggest that increased *A* in post‐anthesis would help facilitate the attainment of the potential maximum individual GW, especially if GN is increased (Figure 2, scenario c). GW can rely on three main sources of assimilates: leaf photosynthesis, spike photosynthesis and the remobilization of the water‐soluble carbohydrates (WSC) from the stem. While efforts have largely focused on selecting and screening for post‐anthesis leaf photosynthetic duration (Blake *et al.*, 2007) and WSC concentration (Rebetzke *et al.*, 2008), spike photosynthesis is an unexplored determining component contributing to GW. When compared with the flag leaf, the spike has shown a higher degree of drought tolerance (Tambussi *et al.*, 2005, 2007) generally explained by a greater intrinsic water‐use efficiency (driven by a low *g_s_* per unit area and a high degree of re‐fixed respiratory CO_2_) and a more pronounced osmotic adjustment (Tambussi *et al.*, 2005, 2007). This situation suggests that spike photosynthesis has an important role in times of water limitation, possibly compensating the flag leaf during grain filling. Furthermore, the assimilates produced in the spike are directly translocated into the grains (Carr and Wardlaw, 1965) leading to a contribution to GW between 10 and 45% depending on environmental conditions and genotype tested (Maydup *et al.*, 2010; Sanchez‐Bragado *et al.*, 2016). Indeed a large variation in gross spike *A* (calculated as the sum of *A* and dark respiration (R_d_) as a proxy of respiration in the light) has been shown in both durum and bread wheat (Maydup *et al.*, 2010; Molero *et al.*, 2013; Zhou *et al.*, 2016; Sanchez‐Bragado *et al.*, 2016), suggesting the existence of natural genetic diversity for exploitation. For instance, the presence of awns (lemma‐derived organs) has been considered an important source of external CO_2_ assimilation of the spike (Maydup *et al.*, 2010) although other factors such a spike morphology (e.g. photosynthetic surface area of spikelets) seems to drive the observed variation in spike *A* (Guo and Schnurbusch, 2016). Earlier evidence proposes that, in the UK, a significant genotypic variation for spike gross *A* and for the contribution of spike *A* to GW is present (Faralli *et al.*, 2019c) and confirms the importance of spike photosynthetic CO_2_ assimilation for grain filling. Additional work is needed to fully understand the underlying mechanism of spike *A,* as well as the extent of existing natural variation. Further development of high‐throughput phenotyping tools focusing on spike *A* would take full advantage of this unexploited trait for GW improvement.

## Conclusion

Photosynthesis is a key determinant of crop yield. Large natural variation in *A* and *A*‐determining traits in different photosynthetic organs exists in a number of crop species that represent a currently unexploited target for crop improvement. Owing to the complexity of the relation between *A* and yield, improvements in high‐throughput, reliable and relevant methodologies will enable the dissection of useful genetic targets for marker‐assisted selection. In wheat, enhancing leaf canopy photosynthesis will increase GN although greater yield will only be achieved with a parallel increase in GW, which relies primarily on enhanced spike photosynthesis. With this in mind, screening for high photosynthetic capacity in both organs should be considered a prime target for high yielding wheat cultivars. In summary, genetic manipulation and elevated [CO_2_] experiments have shown a yield advantage when photosynthesis is increased in food crops; therefore exploiting natural genetic variation in photosynthesis will facilitate the development of cultivars with greater yield potential.

## Data Availability

All data relevant to this review can be found within the manuscript and any supplementary materials if supplied.
